# UPP1 and AHSA1 as emerging biomarkers and targets in pancreatic cancer: A proteomic approach

**DOI:** 10.17305/bb.2025.11958

**Published:** 2025-05-09

**Authors:** Kongfan Zhu, Hua Hu, Yuanfa Tao, Zhijian Yang, Hanjun Li

**Affiliations:** 1 Department of Pancreatic Surgery, Renmin Hospital of Wuhan University, Wuhan, China; 2 Department of Bone and Joint Surgery, Renmin Hospital of Wuhan University, Wuhan, China

**Keywords:** Pancreatic cancer, PC, proteomics, differentiated pancreatic cancer, target, experimental validation

## Abstract

The specific protein targets involved in pancreatic cancer (PC) pathogenesis and its varying levels of differentiation remain incompletely understood. Advanced proteomic methodologies provide a powerful means of identifying key regulatory proteins and signaling pathways central to cancer progression. In this study, proteomic analyses were performed on PC tissue samples of different differentiation grades, along with adjacent non-cancerous (para-PC) tissues. Bioinformatics techniques were used to identify differentially expressed proteins (DEPs) and their associated pathways. Key target proteins were validated using the Gene Expression Profiling Interactive Analysis (GEPIA) database, reverse transcription-quantitative polymerase chain reaction (RT-qPCR), Western blotting, immunohistochemistry (IHC), and immunofluorescence (IF). A total of 431 DEPs were identified between PC and para-PC tissues, while 470 DEPs distinguished poorly differentiated (PD) from moderately differentiated (MD) PCs. Functional enrichment analysis revealed that these DEPs participate in various biological processes and signaling pathways. Five DEPs were common to both comparisons, with Uridine Phosphorylase 1 (UPP1), Lactamase Beta, and Activator of HSP90 ATPase Activity 1 (AHSA1) showing particularly notable differences. UPP1 and AHSA1 were significantly upregulated in PC tissues relative to adjacent tissues and exhibited even higher expression in PD-PCs compared to MD ones. These findings were consistently supported by GEPIA, RT-qPCR, Western blotting, IHC, and IF analyses. This study identifies UPP1 and AHSA1 as key proteins linked to PC differentiation and progression, highlighting their potential as diagnostic markers and therapeutic targets. These insights enhance our understanding of the molecular mechanisms underlying PC and open new avenues for precision treatment strategies.

## Introduction

Pancreatic cancer (PC) is an aggressive and deadly malignancy, projected to become the second leading cause of cancer-related deaths by 2030 [1]. Despite advances in detection and treatment, the five-year survival rate remains below 10%, placing PC among the cancers with the poorest prognosis [2]. PC is histologically classified as well-differentiated (WD), moderately differentiated (MD), or poorly differentiated (PD), with PD-PC exhibiting the greatest invasiveness and worst outcomes [3]. Globally, the incidence of PC continues to rise [4]. Major risk factors include tobacco use, obesity, impaired glucose metabolism, and chronic pancreatitis [5]. At the molecular level, KRAS mutations—present in about 90% of patients—are a key driver of PC [6], along with common alterations in TP53, CDKN2A, and SMAD4 [7]. Although these mutations may differ by tumor grade, the relationship between genetic alterations and PC differentiation remains poorly understood. Early detection of PC remains a major challenge. Currently, dual-phase contrast-enhanced CT is the gold standard for imaging, while MRI may be beneficial for high-risk populations [8]. However, the development of more sensitive and specific biomarkers for early diagnosis remains an urgent unmet need [9]. Proteomics, a rapidly advancing field, provides powerful tools for exploring cancer biology and identifying novel therapeutic targets. Recent studies underscore the potential of proteomics in oncology. For instance, Yang et al. [10] identified KDM1A as a therapeutic target in early-stage esophageal squamous cell carcinoma (ESCC) using a multi-omics approach. Nam et al. [11] found that 2-aminoethanethiol dioxygenase (ADO) may serve as a prognostic marker and therapeutic target in PC. Similarly, Feng et al. [12] used proteomics to uncover a role for fucosyltransferase in ESCC progression, highlighting a new therapeutic avenue. In PC-specific research, Mercanoglu et al. [13] utilized PNA lectin enrichment and mass spectrometry to study the role of GalNT2-catalyzed O-linked glycosylation in pancreatic tissue development, offering new insights into PC pathogenesis. Maebashi et al. [14] employed proteomic techniques to show that methionine restriction inhibits PC proliferation via suppression of the JAK2/STAT3 pathway, revealing potential therapeutic implications. Bruciamacchie et al. [15] combined single-cell and spatial proteomics to demonstrate how ATR inhibitors enhance the efficacy of FOLFIRINOX by remodeling the tumor microenvironment, suggesting new strategies to improve PC treatment. Despite these advancements, several challenges remain. The molecular mechanisms distinguishing PD-PC from MD- and WD-PC are not well understood. Furthermore, while proteomics has shown great promise in cancer research, its clinical translation remains limited. To address these gaps, we propose the following hypothesis: proteomic analysis can uncover molecular differences between PD-PC and MD-PC, identifying key regulatory proteins and signaling pathways involved in tumor differentiation. This research aims to clarify the molecular basis of PC differentiation and its link to prognosis, ultimately facilitating the discovery of novel diagnostic markers and therapeutic targets. Our proposed methodology includes several key steps. First, PC tissue samples representing different differentiation grades will be collected for proteomic profiling. Advanced bioinformatics tools—including Weighted Gene Co-expression Network Analysis (WGCNA) and the Mfuzz clustering algorithm—will be applied to identify critical protein modules and regulatory networks associated with tumor grade. The clinical relevance of these proteins will then be assessed through integration with public datasets and experimental validation. This study holds significant scientific and clinical value. It will address critical gaps in our understanding of PC differentiation, provide a detailed molecular profile of tumors across differentiation grades, and identify novel biomarkers and therapeutic targets. Ultimately, these insights may support the development of precision medicine strategies for PD-PC.

## Materials and methods

### Patients

This study involved 22 patients with PDAC recruited from Hubei Provincial People’s Hospital. Tissue biopsy samples were collected surgically and immediately placed in a refrigerated sterile preservation solution on ice packs. After surgery, the samples were accurately labeled and maintained at a controlled temperature during transport. For long-term preservation, samples were either fixed in 10% formalin solution or stored in a −80 ^∘^C freezer. Of the collected tissues, eight PD-PC and seven MD-PC samples were used for proteomic analysis, while five PC samples were used for reverse transcription-quantitative polymerase chain reaction (RT-qPCR) and Western blot assays. Additionally, one PD-PC and one MD-PC sample were used for immunohistochemistry (IHC) experiments. The clinical characteristics of the patients are summarized in Table 1.

**Table 1:** Clinical characteristics of the patients involved in the study

**Characteristic**	**Levels**	**Overall**
Tumor size	386 ± 1443 cm^3^	
Location	Head and neck	13
	Body and tail	9
*Tumor differentiation degree*	Poor	10
	Moderate	12
*T stage, n(%)*	T1	3
	T2	6
	T3	9
	T4	4
*N stage, n(%)*	N0	10
	N1	5
	N2	5
	NX	2
*M stage, n(%)*	M0	15
	M1	7
*Bile duct infiltration*	YES	5
	NO	17
*Duodenal infiltration*	YES	5
	NO	17
*Perineural invasion*	YES	19
	NO	3
*Vascular invasion*	YES	10
	NO	12
*Gender, n(%)*	Female	8
	Male	14
*Age, n(%)*	<=65	12
	>65	10
*Lymph node metastasis*	YES	12
	NO	10
*Liver metastasis*	YES	3
	NO	19
CEA	5.64 ± 14.4 ng/mL	
CA19-9	1806 ± 2533 U/mL	
*Vital status*	Live	14
	Dead	8

### Proteomic analysis

#### Analysis process

Protein profiling analysis involved detecting the range of peptides in different samples, mapping the identified peptide fragments to their corresponding protein sequences, and quantifying the number of proteins. To assess the accuracy of the profiling results, we examined the raw mass spectrometry data and calculated the average number of peptides traced to each protein. To ensure data quality, we retained only the 7635 proteins present in more than 50% of samples in at least one group. Missing values for each protein were imputed using multivariate normal imputation (MVNI). Pearson’s correlation coefficient and principal component analysis (PCA) were used to evaluate intra- and inter-group differences. Proteomic features were identified by analyzing proteins with varying fold changes (FC) across groups. Differentially expressed proteins (DEPs) were defined using the criteria |log_2_(FC)| > 0.263 and unpaired *P* < 0.05, and were visualized using volcano plots and hierarchical clustering heatmaps. Gene set enrichment analysis (GSEA), Gene Ontology (GO), and Kyoto Encyclopedia of Genes and Genomes (KEGG) pathway analyses were performed to explore the signaling pathways associated with DEPs. Protein–protein interaction (PPI) networks were constructed to investigate relationships among DEPs. Mfuzz analysis was used to identify trends in DEP expression, and WGCNA was applied to identify modules enriched with differential proteins.

#### Sample preparation

The samples were mixed with 8 M urea and 100 mM Tris-Cl, then subjected to water bath sonication. After centrifugation, the protein concentration of the supernatant was measured using the BCA method. Protein reduction and alkylation were performed with TCEP and CAA at 37 ^∘^C for 1 h. Urea was then diluted to below 2 M using 100 mM Tris-HCl (pH 8.0). Trypsin was added at an enzyme-to-protein ratio of 1:50 (w/w) for overnight digestion at 37 ^∘^C. The following day, TFA was added to adjust the pH to 6.0 to terminate the digestion. After centrifugation (12,000 × *g*, 15 min), the supernatant was subjected to peptide purification using a self-made SDB-RPS desalting column. The peptide eluate was vacuum-dried and stored at −20 ^∘^C for later use.

#### Mass spectrometry analysis

All samples were analyzed using a timsTOF Pro (Bruker Daltonics), a hybrid trapped ion mobility spectrometry (TIMS) quadrupole time-of-flight mass spectrometer. An UltiMate 3000 RSLCnano system (Thermo) was coupled to the timsTOF Pro via a CaptiveSpray nano ion source (Bruker Daltonics). Peptide samples were injected into a C18 trap column (75 µm × 2 cm, 3 µm particle size, 100 Å pore size, Thermo) and separated on a reversed-phase C18 analytical column (75 µm × 15 cm, 1.7 µm particle size, 100 Å pore size, IonOpticks). Mobile phase A (0.1% formic acid in water) and mobile phase B (0.1% formic acid in acetonitrile) were used to generate a separation gradient at a flow rate of 300 nL/min. The mass spectrometer was operated in diaPASEF mode. The capillary voltage was set to 1500 V. MS and MS/MS spectra were acquired over a range of 100–1700 m/z. Ion mobility was scanned from 0.6 to −1.6 Vs/cm^2^. Accumulation and ramp times were both set to 50 ms. The diaPASEF acquisition scheme was defined in the m/z–ion mobility plane using tims Control software (Bruker Daltonics). Collision energy was ramped linearly as a function of mobility, from 59 eV at 1/K_0_ ═ 1.6 Vs/cm^2^ to 20 eV at 1/K_0_ ═ 0.6 Vs/cm^2^.

#### Peptide and protein identification and quantification

DIA raw data were analyzed using DIA-NN (v1.8.1). Spectra files were searched against the human protein sequence database (June 19, 2023; 20,423 entries) downloaded from UniProt. A library-free search was performed according to the DIA-NN manual (https://github.com/vdemichev/DiaNN/). A predicted in silico spectral library was generated from the FASTA database. Specific Trypsin/P was selected as the digestion method, allowing one missed cleavage. Carbamidomethylation on cysteine residues was set as a fixed modification, while oxidation of methionine and acetylation of protein N-termini were set as variable modifications. The false discovery rate (FDR) was set to 0.01 for reliable precursor identification. “MBR” and “heuristic protein inference” options were enabled. Protein intensities were normalized using the MaxLFQ algorithm.

#### Data analysis

For DIA data quantification, the output files generated by DIA-NN for each sample were processed in the R workspace and used for downstream analysis. Intensity values were log2-transformed prior to statistical evaluation. To ensure data quality and maximize the utility of the proteomic data, proteins with more than 50% missing values within each group were excluded. Missing values were imputed using a MVNI approach, simulating values from a normal distribution around the mass spectrometer’s detection limit. Specifically, the mean and standard deviation of the observed intensity distribution were first calculated, and a new distribution was generated by applying a downshift of 1.8 standard deviations and a width of 0.25 standard deviations. This distribution was then used to impute missing values across the entire matrix, enabling further statistical analysis. To identify DEPs between groups in our DIA-MS-based proteomics study, unpaired *t*-tests were performed. Proteins with *P* < 0.05 and an FC >1.2 or <1/1.2 were considered significantly different. Functional enrichment analysis of quantified proteins was conducted using GO, the KEGG, and Hallmark gene sets. Fisher’s exact test was used to compare DEPs against background proteins. GO or KEGG terms with *P* ≥ 0.05 and at least three proteins were considered significantly enriched among DEPs. GSEA was also performed, and significant gene sets were identified using the following criteria: normalized enrichment score (NES) > 1, nominal *P* value < 0.01, and FDR *q* value < 0.25. Finally, PPI networks were generated using the STRING database (https://string-db.org/), retaining only interactions with a combined score> 0.4.

### Gene Expression Profiling Interactive Analysis (GEPIA) database analysis

We used the GEPIA database to examine the expression levels of Uridine Phosphorylase 1 (UPP1), Lactamase Beta (LACTB), and Activator of HSP90 ATPase Activity 1 (AHSA1) in PC tissues. Expression data were extracted from 179 PC samples and 171 adjacent non-cancerous (para-PC) tissue samples for comparative analysis. Protein expression levels were quantified using the standard protocols integrated into the GEPIA platform. Differential gene expression analysis was performed using the limma package in R with default settings. Statistical significance was assessed using Student’s *t*-test, with 0.05 considered significant. Box plots were generated to visualize protein expression differences between PC and para-PC groups. All analyses were conducted using GEPIA’s integrated tools, and results were further processed and visualized in R (version 4.0.3, R Foundation for Statistical Computing) using the ggplot2 package for improved graphical presentation.

### WGCNA

We obtained RNA-seq data and corresponding clinical information for PC patients from The Cancer Genome Atlas (TCGA) database. Raw count data were normalized using the DESeq2 package (Bioconductor) and log_2_-transformed. Genes with low expression (counts <10 in more than 80% of samples) were filtered out. DEGs between tumor and normal samples were identified using the limma package (Bioconductor), with |log_2_FC| > 1 and an adjusted *P* value <0.05 as the cutoff criteria. We then constructed a gene co-expression network using the WGCNA package in R. A soft-thresholding power was selected to approximate scale-free topology. The topological overlap matrix (TOM) was calculated and used for hierarchical clustering to identify gene modules.

### Mfuzz analysis

For protein expression analysis, we used the Mfuzz package (version 2.48.0) in R (version 4.0.2) to perform soft clustering. Raw protein expression data were log2-transformed and standardized using the standardize function in Mfuzz. Proteins were clustered based on their expression patterns across the para-PC, PD-PC, and MD-PC groups using the mfuzz function, with the number of clusters (c) set to six and the fuzzifier parameter (m) set to 2.5. These parameters were optimized using the mestimate function. DEPs were identified using the limma package, applying an FDR < 0.05 and an FC > 1.5. Mfuzz cluster plots were generated using the mfuzz.plot function.

### RT-qPCR

Total RNA was extracted from tissue using TRIzol reagent (Ambion, 15596-026) and reverse-transcribed into cDNA with HiScript II Select qRT SuperMix II (VAZYME, R233). RT-qPCR was then performed using AceQ qPCR SYBR Green Master Mix (VAZYME, Q111) and the corresponding primer sequences (Table 2). Data were analyzed using the 2^−ΔΔCt^ method.

**Table 2:** Primer sequences

**Name**	**Primer**	**Sequence**	**Size**
*Homo β-actin*	Forward	CCCTGGAGAAGAGCTACGAG	180 bp
	Reverse	CGTACAGGTCTTTGCGGATG	
*Homo UPP1*	Forward	TTCTGGTGGGATAGGTCTGG	168 bp
	Reverse	AGCTCTGCAGAACACAGCAA	
*Homo LACTB*	Forward	TATGTTCCCGAATTCCCAGA	249 bp
	Reverse	TTTGGCTTCATTCTCCTGCT	
*Homo AHSA1*	Forward	CAGCCAGCACTGAAAACTGA	164 bp
	Reverse	ACCAGCTCTTGGGTGGTAAA	

UPP1: Uridine Phosphorylase 1; LACTB: Lactamase Beta; AHSA1: Activator of HSP90 ATPase Activity 1.

### Western blot

For protein extraction, tissue samples were placed in EP tubes containing RIPA lysis buffer (Servicebio) and homogenized using an automatic grinder (Tissvelyser-24L, Shanghai Jingxin). After centrifugation, the supernatant was collected, and protein concentrations were determined using the BCA method (Guangzhou JeBest Biotechnology). Forty micrograms of each protein sample were mixed with loading buffer and heated at 95 ^∘^C for 10 min. Proteins were then separated by SDS-PAGE using 12% separating and 5% stacking gels. Following electrophoresis, proteins were transferred to PVDF membranes (Millipore). Membranes were blocked with 5% non-fat milk in TBST for 2 h, then incubated overnight at 4 ^∘^C with primary antibodies. The primary antibodies used were UPP1 (Wuhan Sanying Biotechnology, 1:2000), LACTB (Boster, 1:2000), AHSA1 (Boster, 1:2000), and GAPDH (Affinity, 1:20,000). After washing with TBST, membranes were incubated with appropriate HRP-conjugated secondary antibodies (Beyotime Biotechnology) for 2 h at room temperature. Chemiluminescent detection was performed using ECL substrate (Servicebio), and membranes were exposed to X-ray film. Protein band intensities were analyzed using Image-Pro Plus software, with GAPDH serving as the internal control for normalization.

### Tissue sections

Tissue samples from eight PD-PC and seven MD-PC cases were processed for histological examination. Samples were fixed in 10% neutral buffered formalin, followed by dehydration through a graded alcohol series. Specimens were then cleared with xylene and embedded in paraffin blocks. Using a Leica RM2016 microtome, 4 µm-thick sections were cut and mounted onto microscope slides. These sections were used for hematoxylin and eosin (H&E) staining, immunofluorescence (IF), and IHC.

### H&E staining

For H&E staining, tissue sections were deparaffinized in xylene, rehydrated through a graded ethanol series, and stained with Mayer’s hematoxylin (BT-P107, Qisai Biological) for 5 min, followed by 1% eosin Y (BT-P109, Qisai Biological) for 5 min. After staining, sections were dehydrated, cleared, and mounted with neutral balsam. Images were acquired using a Leica FLEXACAM C1 microscope equipped with LAS X imaging software at 200× magnification. This protocol enabled clear visualization of nuclear (blue) and cytoplasmic (pink to red) structures, facilitating detailed morphological analysis of PD-PC and MD-PC tissues.

### IF

For IF, tissue sections were deparaffinized, rehydrated, and subjected to heat-induced antigen retrieval using Tris-EDTA buffer (pH 9.0, Qisai Biological) at 95 ^∘^C for 15 min. Multiplex IF staining was performed using a tyramide signal amplification system. Primary antibodies against UPP1 (1:100, Sanying), AHSA1 (1:100, Boster), and LACTB (1:100, Boster) were applied sequentially, followed by HRP-conjugated secondary antibodies and tyramide-fluorophore labeling. Between each staining round, microwave treatment was used to strip the previous antibodies while preserving the fluorescent signal. Nuclei were counterstained with DAPI (Beyotime). Images were captured using a multispectral imaging system built on an Olympus BX53 fluorescence microscope platform, with at least three representative fields acquired per sample at 200× magnification. Image analysis was conducted using cellSens Entry software (Olympus) to quantify the fluorescence intensity of each marker. Statistical analysis was performed to compare protein expression levels between PD-PC and MD-PC samples.

**Figure 1.**
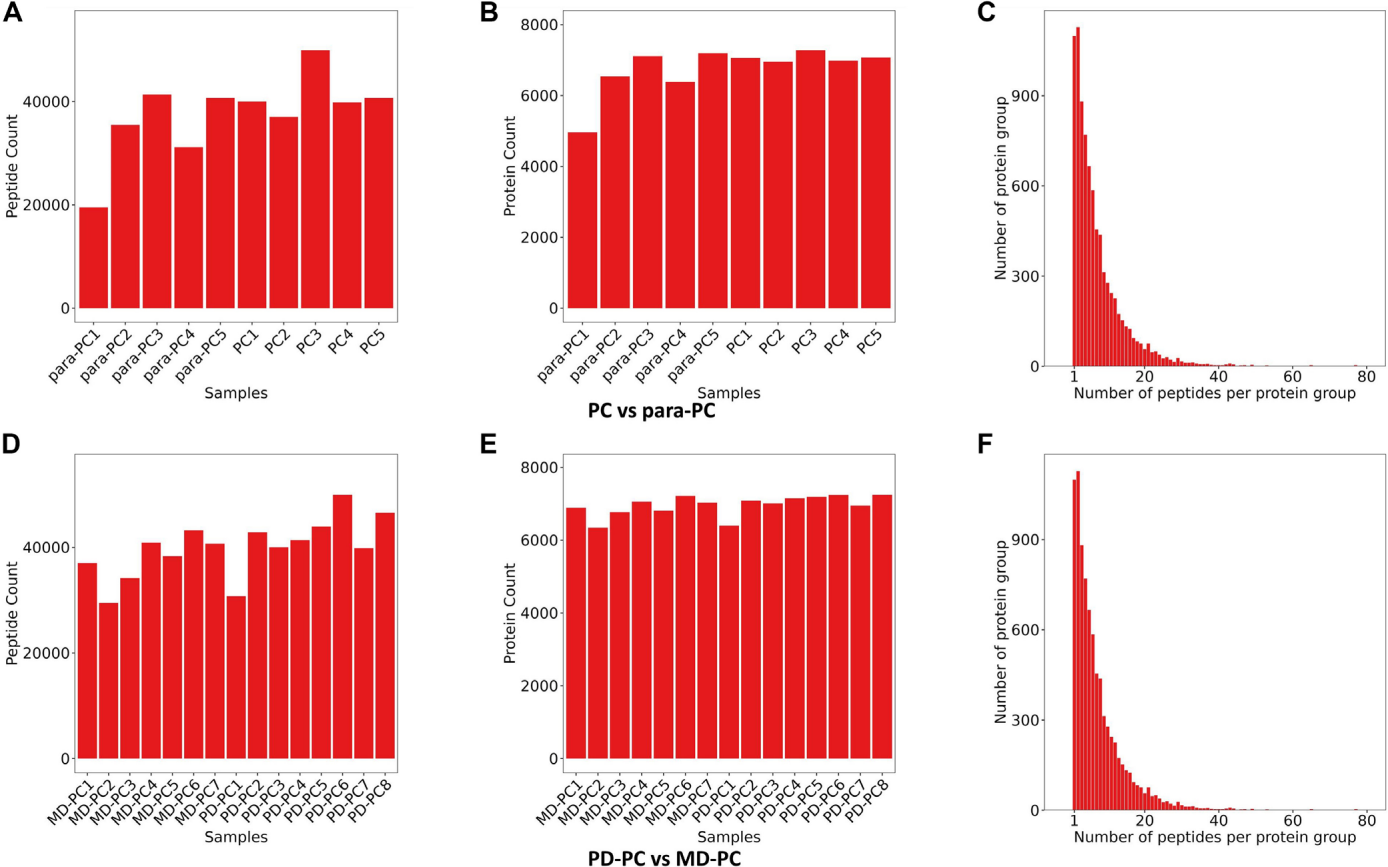
**Proteomics analysis overview.** (A and D) The distribution of numbers of quantified peptides in detected samples; (B and E) The distribution of numbers of quantified proteins in detected samples; (C and F) The distribution of peptide numbers of quantified proteins. PD: Poorly differentiated; PC: Pancreatic cancer; MD: Moderately differentiated.

### IHC

For IHC, tissue sections were subjected to antigen retrieval using Tris-EDTA buffer (pH 9.0) in a pressure cooker for 15 min. Endogenous peroxidase activity was blocked with 3% hydrogen peroxide (H_2_O_2_). Sections were incubated overnight at 4 ^∘^C with primary antibodies against UPP1 (1:100, Proteintech), AHSA1 (1:100, Boster), and LACTB (1:100, Boster). After washing, sections were treated with an HRP-conjugated secondary antibody (Dako) for 30 min at 37 ^∘^C. Immunoreactivity was visualized using DAB (Dako), followed by hematoxylin counterstaining. The stained sections were then dehydrated, cleared, and mounted. Micrographs were acquired using a BX53 optical microscope (Olympus) equipped with cellSens Entry software. Protein expression was quantified by measuring the average optical density of positively stained areas using ImageJ software. All staining procedures were performed in triplicate to ensure reproducibility.

### Survival analysis

Using the TCGA dataset, patient samples were stratified into low- and high-expression groups for the genes LACTB, UPP1, and AHSA1, based on optimal cutoff values determined by the survey_cutpoint function from the surveyor package in R (version 4.4.1). These cutoff values were calculated using the Youden index (sensitivity + specificity – 1), which identifies the threshold that best separates the groups by maximizing the index. This approach enhances group differentiation while minimizing the *P* value in Kaplan–Meier survival analysis [16].

### Ethical statement

This study was approved by the Ethics Committee of Hubei Provincial People’s Hospital (Approval No. WDRY2024-K188) and conducted in accordance with the Declaration of Helsinki. Informed consent was obtained from all individual participants.

### Statistical analysis

Prism software was used to perform statistical analyses for experimental verification. We employed Student’s *t*-test to evaluate differences between pairs of cohorts. A *P* value of <0.05 was considered statistically significant.

## Results

### Proteomics analysis overview

**Figure 2.**
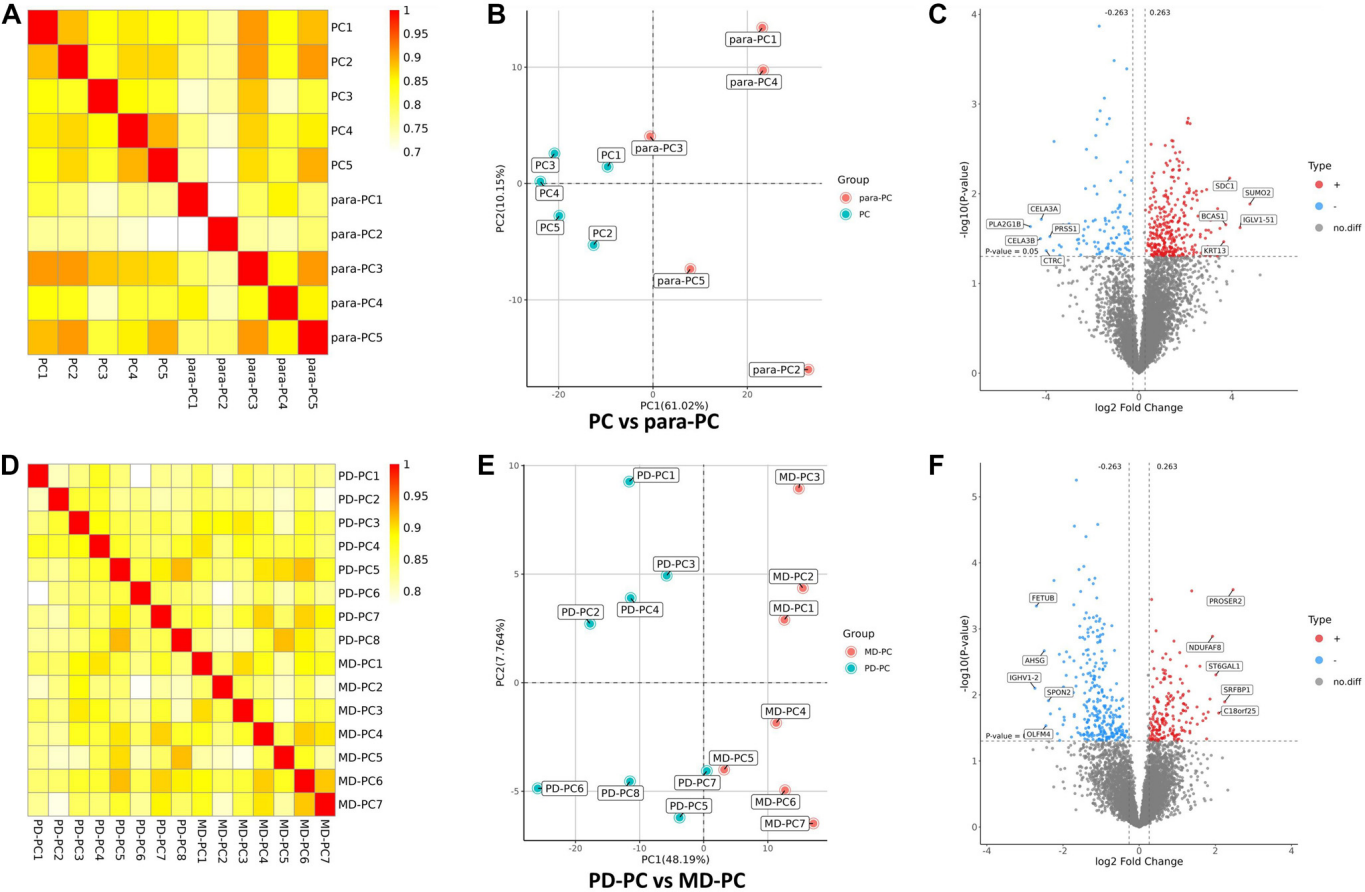
**Protein difference analysis.** (A and D) Pearson’s correlation of protein quantitation; (B and E) Sample repeatability analysis by principal component analysis; (C and F) Volcano plot showing the differentially expressed proteins. GO: Gene Ontology; BP: Biological processes; PD: Poorly differentiated; PC: Pancreatic cancer; MD: Moderately differentiated.

We conducted two proteomic studies. The first compared five PC tissue samples—three PD and two MD—with adjacent non-cancerous (para-PC) tissues. The second study compared eight PD-PC tissue samples with seven MD-PC tissue samples. These analyses identified 64,697 and 65,505 peptides, corresponding to 8307 and 8340 human proteins, respectively. The reliability of our results was confirmed by examining the correlation between peptide and protein counts (Figure 1). In the PC vs para-PC group, Pearson correlation coefficients exceeded 0.7, and PCA revealed clear separation between the PC and para-PC samples, with PC samples forming a tight cluster. A total of 431 DEPs were identified (Table S1), including 332 upregulated and 99 downregulated DEPs (Figure 2A–2C). Similarly, in the PD-PC vs MD-PC group, intra-group Pearson correlation coefficients exceeded 0.8. PCA showed distinct separation between PD-PC and MD-PC samples along the PC1 axis, indicating substantial differences in protein expression. In this comparison, 470 DEPs were identified (Table S2), with 180 proteins upregulated and 290 downregulated (Figure 2D–2F).

**Figure 3.**
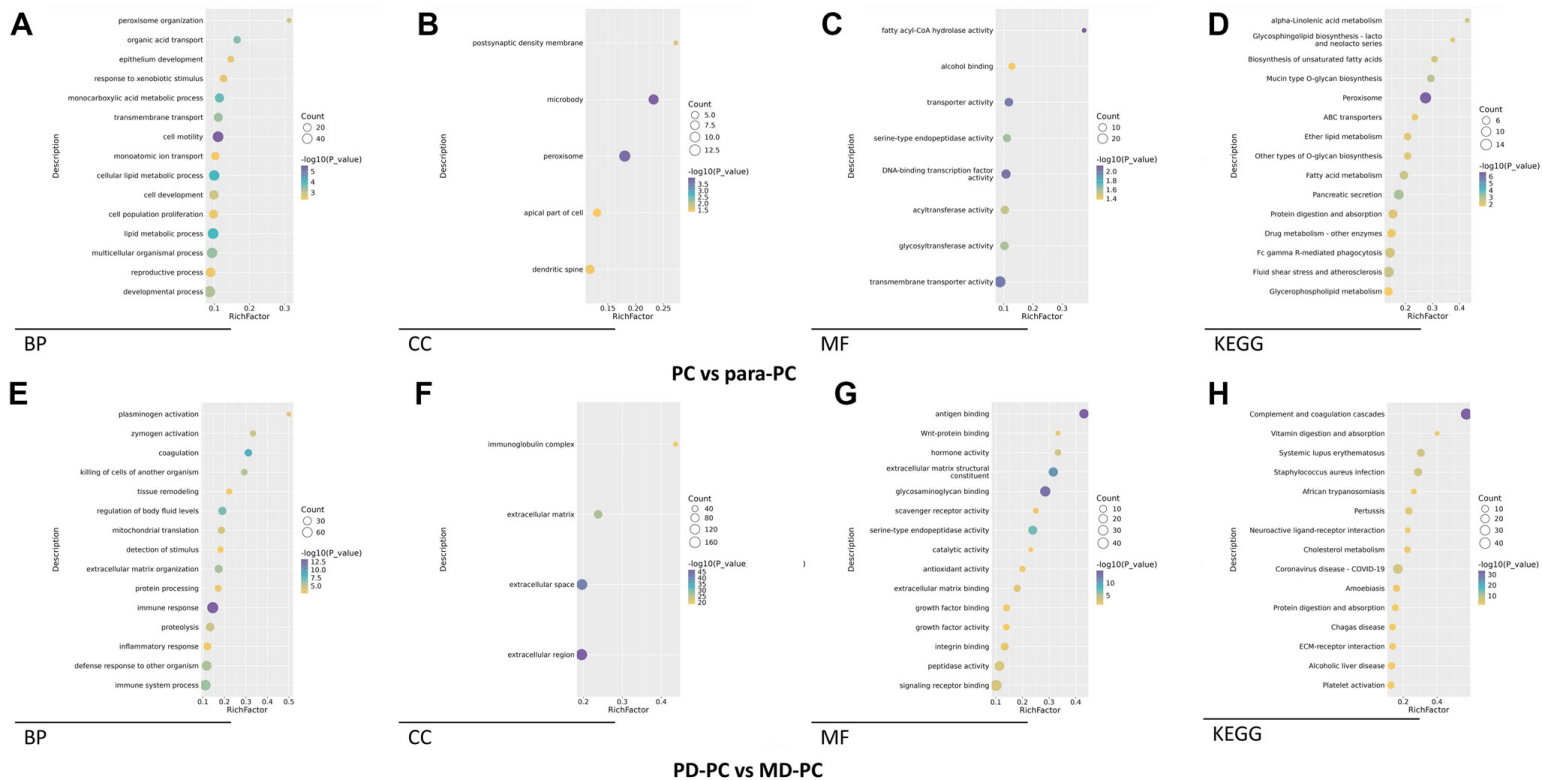
**GO and KEGG enrichment analysis of DEPs.** (A and E) GO-based enrichment analysis of DEPs shown in the term of BP; (B) and (F) GO-based enrichment analysis of DEPs shown in the term of CC; (C and G) GO-based enrichment analysis of DEPs shown in the term of MF; (D and H) KEGG-based enrichment analysis of DEPs. PD: Poorly differentiated; PC: Pancreatic cancer; MD: Moderately differentiated; KEGG: Kyoto Encyclopedia of Genes and Genomes; GO: Gene Ontology; DEP: Differentially expressed protein; BP: Biological processes; CC: Cellular component; MF: Molecular function.

### Function enrichment analysis of DEPs

**Figure 4.**
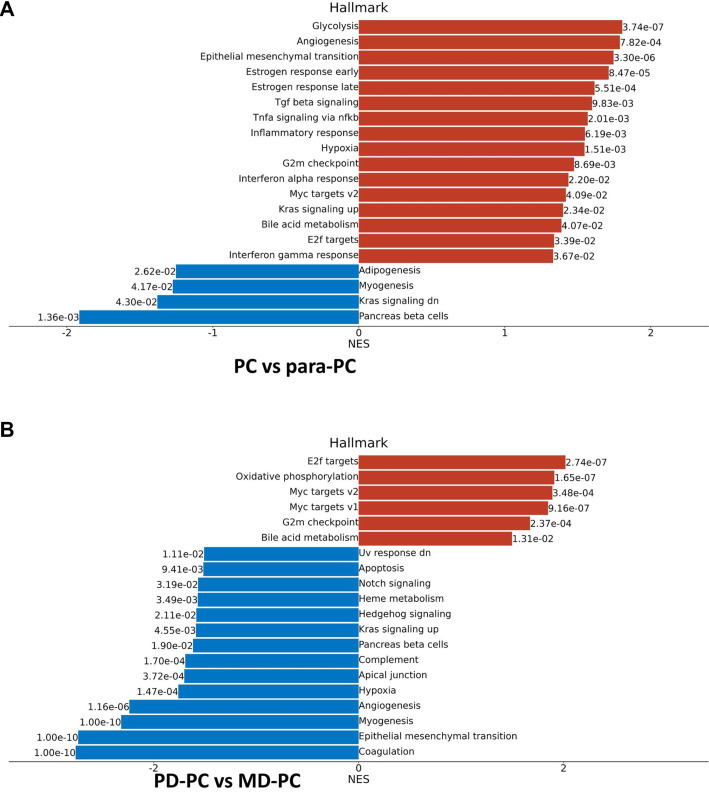
**GSEA of DEPs.** (A) Hallmark gene set-based enrichment analysis by GSEA between PC group and para-PC group; (B) Hallmark gene set-based enrichment analysis by GSEA between PD-PC group and MD-PC group. NES: Normalized enrichment score; PD: Poorly differentiated; PC: Pancreatic cancer; MD: Moderately differentiated; GSEA: Gene set enrichment analysis; NES: Normalized enrichment score.

**Figure 5.**
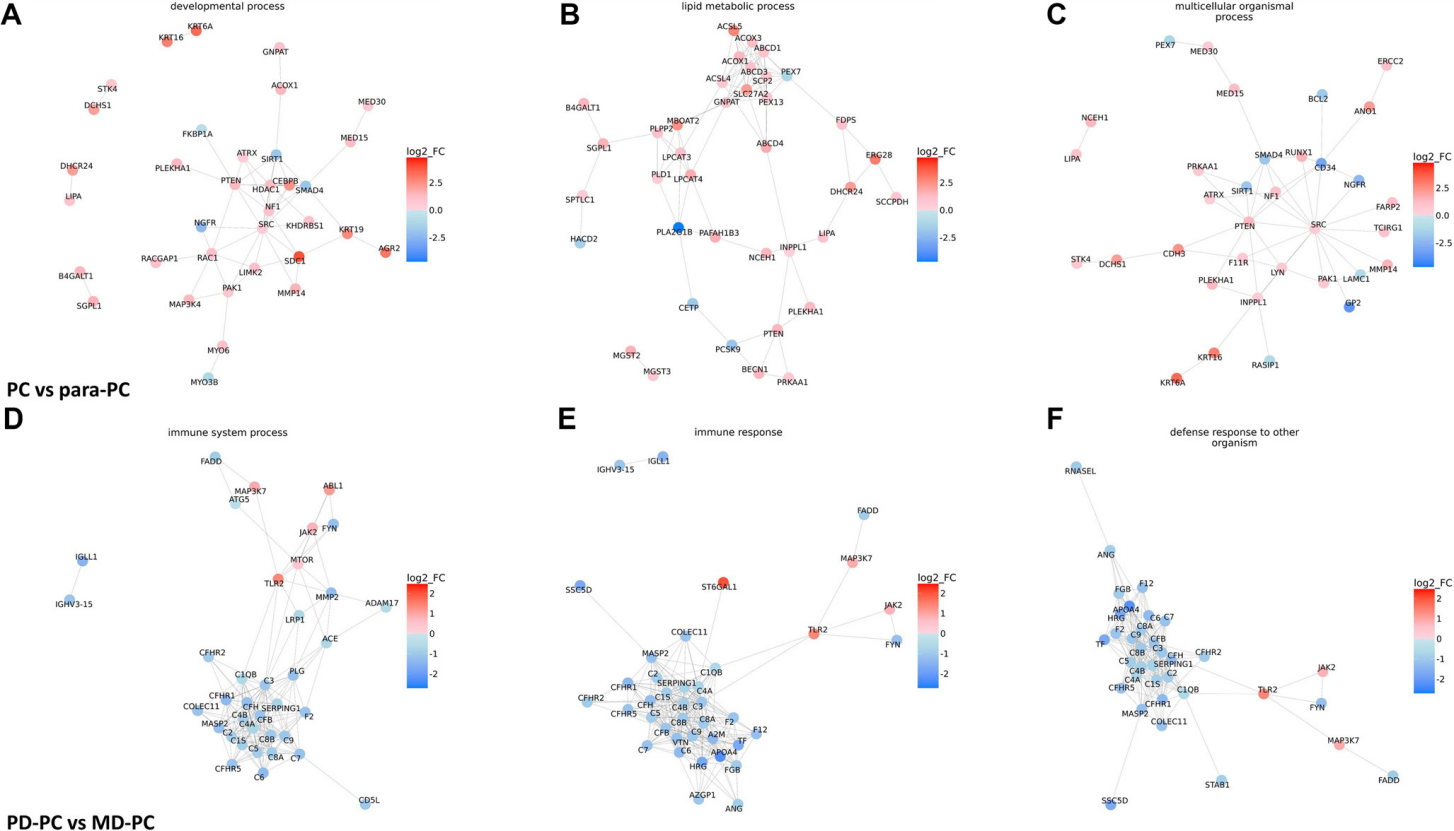
**Protein–protein network analysis of DEPs.** (A) DEPs in developmental process; (B) DEPs in lipid metabolic process; (C) DEPs in multicellular organismal process; (D) DEPs in immune system process; (E) DEPs in immune response; (F) DEPs in defense response to another organism. Relevance network graph depicting the correlation of proteins derived from DEPs using PPI analysis respectively. Circles indicate the protein ID, Line width indicates interaction strength, red indicates up-regulated proteins, and blue indicates down-regulated proteins. The darker the color, the greater the difference. DEP: Differentially expressed protein; PPI: Protein–protein interaction; PD: Poorly differentiated; PC: Pancreatic cancer; MD: Moderately differentiated.

Using GO, KEGG, and GSEA, we conducted pathway and functional analyses of the DEPs. In the PC vs para-PC group, the most significantly enriched biological process (BP) was cell motility, followed by cellular lipid metabolic process and lipid metabolic process (Figures 3A and S1A). The top cellular components (CCs) were microbody and peroxisome (Figures 3B and S1B), while the most significantly enriched molecular functions (MFs) were fatty acyl-CoA hydrolase activity and DNA-binding transcription factor activity (Figures 3C and S1C). Correspondingly, the KEGG analysis identified the peroxisome pathway as the most significant, while Alpha-linolenic acid metabolism and glycosphingolipid biosynthesis (lacto and neolacto series) had the highest rich factors (Figures 3D and S1D). In the PD-PC vs MD-PC group, the most enriched BP was immune response, followed by coagulation and regulation of body fluid levels (Figures 3E and S2). The most enriched CC terms were extracellular region and extracellular space (Figures 3F and S3), while the top MFs were signaling receptor binding and peptidase activity (Figures 3G and S4). In KEGG analysis, the complement and coagulation cascades pathway was the most significantly enriched, followed by ECM-receptor interaction, alcoholic liver disease, and platelet activation (Figures 3H and S5). GSEA Hallmark gene set analysis further revealed that in the PC vs para-PC group, the pathways with the highest normalized enrichment scores (NES) for upregulated DEPs were Glycolysis (*P* ═ 3.74e-07) and Angiogenesis (*P* ═ 7.82e-04), while Pancreas Beta Cells was the most significantly downregulated pathway (NES; *P* ═ 1.36e-03) (Figure 4A). In the PD-PC vs MD-PC group, E2F Targets was the most enriched pathway among upregulated DEPs (*P* ═ 2.74e-07), while epithelial–mesenchymal transition and Coagulation were the most enriched among downregulated DEPs (both *P* ═ 1.00e-10) (Figure 4B). Notably, the Myogenesis pathway appeared in both comparisons, although it did not have the highest NES in either. PPI network construction revealed that in the PC vs para-PC group, the DEPs with the most interactions were SRC (degree ═ 41), PTEN (degree ═ 30), and HDAC1 (degree ═ 25). The BPs with the highest number of protein nodes in the PPI network were developmental process (count ═ 48), lipid metabolic process (count ═ 43), and multicellular organismal process (count ═ 42) (Figure 5A–5C). In contrast, in the PD-PC vs MD-PC group, the top hub proteins were C4B (degree ═ 88), A2M (degree ═ 59), and VTN (degree ═ 58). The most enriched BPs in the network were immune system process (count ═ 46), immune response (count ═ 45), and defense response to other organisms (count ═ 39) (Figure 5D–5F). These results clearly demonstrate that the DEPs and their associated functions differ substantially between the PC vs para-PC and PD-PC vs MD-PC comparisons. However, given the intrinsic connection between differentiated PC and PC, an integrated analysis of PC, PD-PC, and MD-PC is warranted to identify key targets involved in PC progression.

### PC coalition analysis

**Figure 6.**
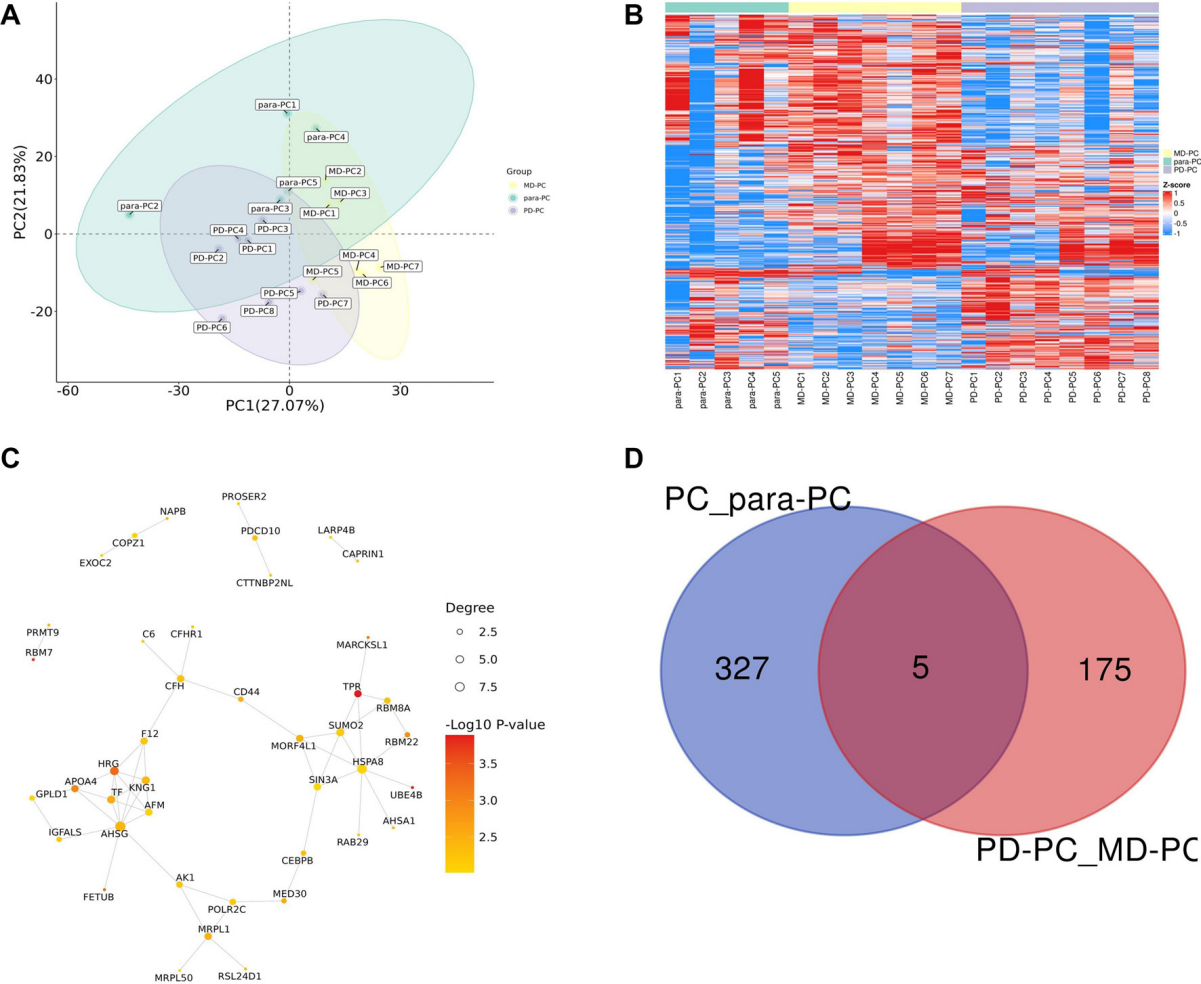
**Integrated Analysis.** (A) Principal Component Analysis (PCA) of DEPs in PC, PD-PC, and MD-PC. (B) Heatmap illustrating quantitative data of DEPs in PC, PD-PC, and MD-PC. Each row represents a protein, while each column represents a sample. The color intensity indicates the quantitative data of individual proteins in corresponding samples, displayed as log2-transformed signal intensities. (C) PPI network of DEPs in PC, PD-PC, and MD-PC groups, analyzed using the STRING database. Each node represents a DEP, with edges between nodes indicating known or predicted PPIs. Node color denotes the fold change or other relevant scores for each protein, while node size can represent the degree of connectivity within the interaction network. (D) Venn diagram depicting the overlap of DEPs between PC vs para-PC group and PD-PC vs MD-PC group. PD: Poorly differentiated; PC: Pancreatic cancer; MD: Moderately differentiated; DEP: Differentially expressed protein; PPI: Protein–protein interaction.

**Figure 7.**
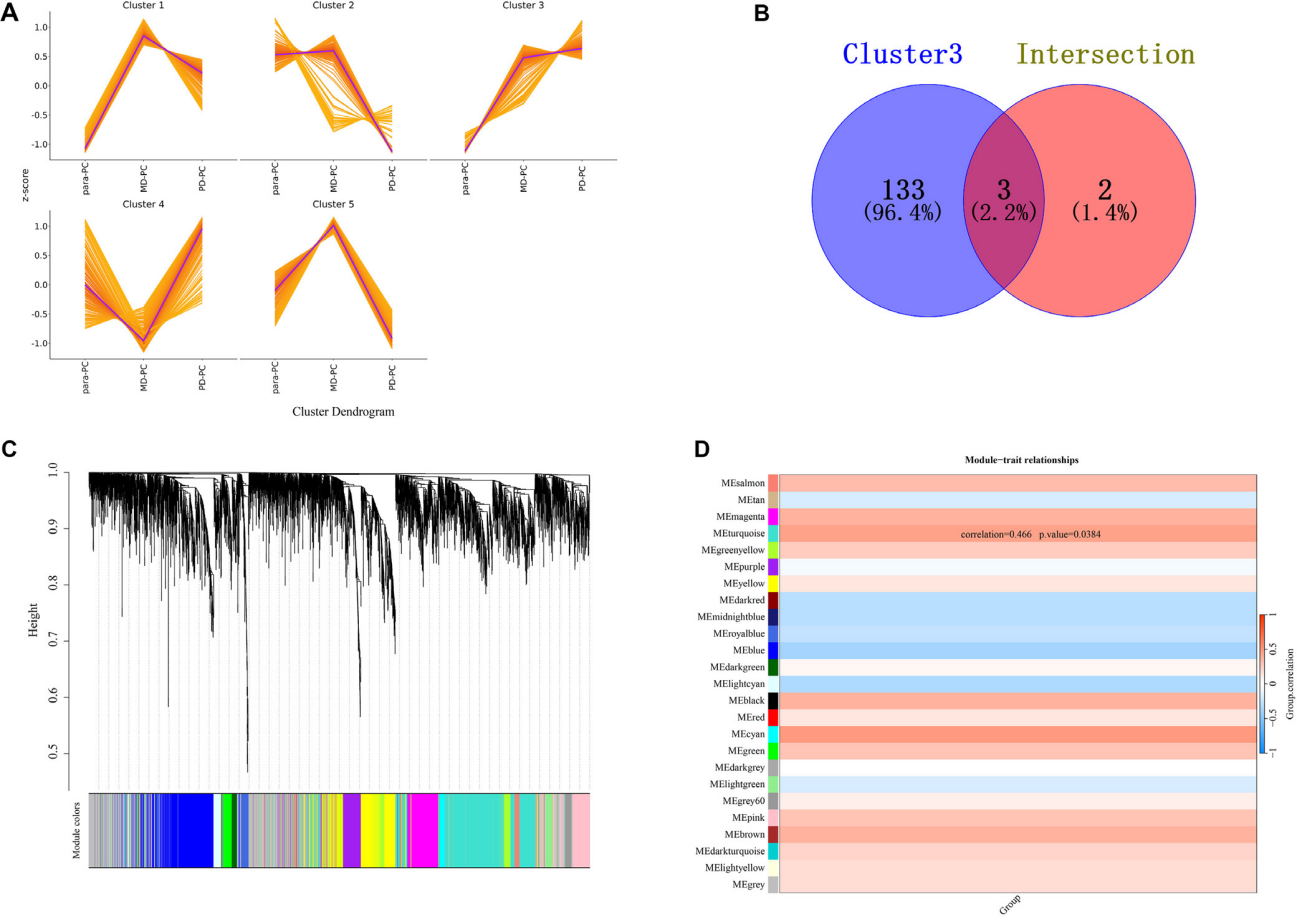
**Analysis using Mfuzz and WGCNA.** (A) Mfuzz clustering of differentially expressed proteins in para-PC, PD-PC, and MD-PC groups. Each graph represents proteins clustered by similar expression patterns. The *x*-axis denotes sample groups, while the *y*-axis indicates relative protein quantification. Each line represents an individual protein, with its shape illustrating the quantitative changes across different sample groups. (B) Venn diagram showing the intersection of five proteins with cluster 3. (C) WGCNA analysis. (D) Correlation analysis of 25 modules. PD: Poorly differentiated; PC: Pancreatic cancer; MD: Moderately differentiated; WGCNA: Weighted Gene Co-expression Network Analysis.

**Figure 8.**
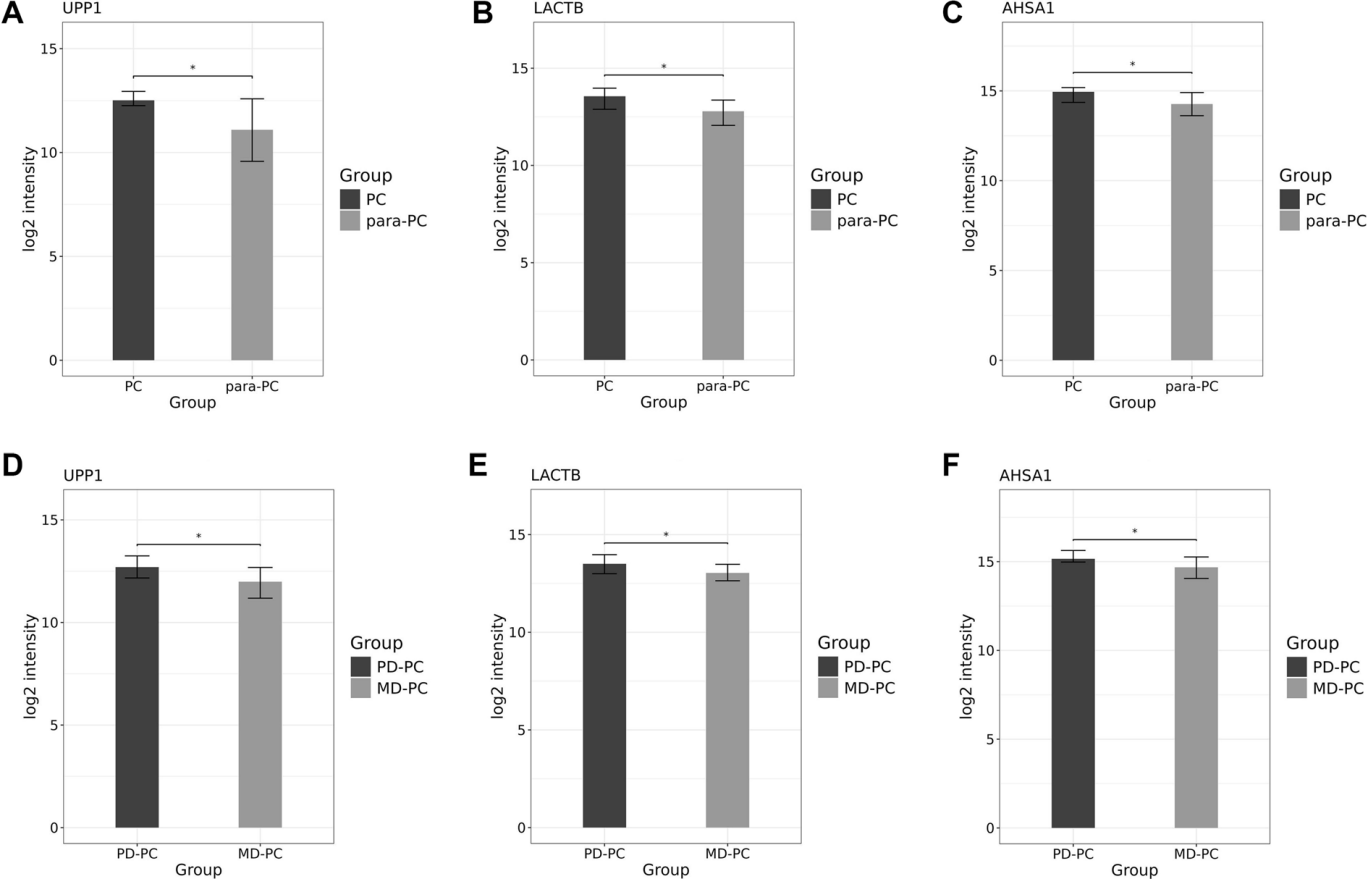
**Quantitative expression profiles of UPP1, LACTB, and AHSA1 proteins in omics analysis.** (A–C) Protein quantification values of UPP1, LACTB, and AHSA1 in PC vs para-PC group; (D–F) Protein quantification values of UPP1, LACTB, and AHSA1 in PD-PC vs MD-PC group. *P < 0.05. PD: Poorly differentiated; PC: Pancreatic cancer; MD: Moderately differentiated; UPP1: Uridine Phosphorylase 1; LACTB: Lactamase Beta; AHSA1: Activator of HSP90 ATPase Activity 1.

Using PCA, clustering heatmap, and PPI network analyses, we identified both common and distinct key proteins among PC, PD-PC, and MD-PC patients (Figure 6A–6C). Ultimately, five DEPs—UPP1, SCYL2, LACTB, AHSA1, and ABHD6—were found to be shared between the PC vs para-PC and PD-PC vs MD-PC comparisons (Figure 6D). Mfuzz analysis of protein expression patterns across para-PC, PD-PC, and MD-PC groups revealed that proteins in Module 3 exhibited a progressively increasing expression trend (Figure 7A). Therefore, subsequent screening of the five DEPs focused on their association with Module 3. This module contained 136 DEPs, and its intersection with the common DEPs from the two group comparisons yielded three overlapping proteins: UPP1, LACTB, and AHSA1 (Figure 7B). WGCNA analysis further clustered all DEPs into 25 modules (Figure 7C), with the three intersecting proteins enriched in the turquoise module. Correlation analysis showed that the turquoise module had a correlation coefficient of 0.47 (*P* ═ 0.038) with other modules, suggesting a potentially critical role in PC initiation and progression (Figure 7D). To further elucidate the significance of UPP1, LACTB, and AHSA1 in PC development, we quantified their expression using proteomic data. As shown in Figure 8, the levels of these proteins were higher in PC compared to para-PC and elevated in PD-PC relative to MD-PC.

### GEPIA database and experimental verification targets

**Figure 9.**
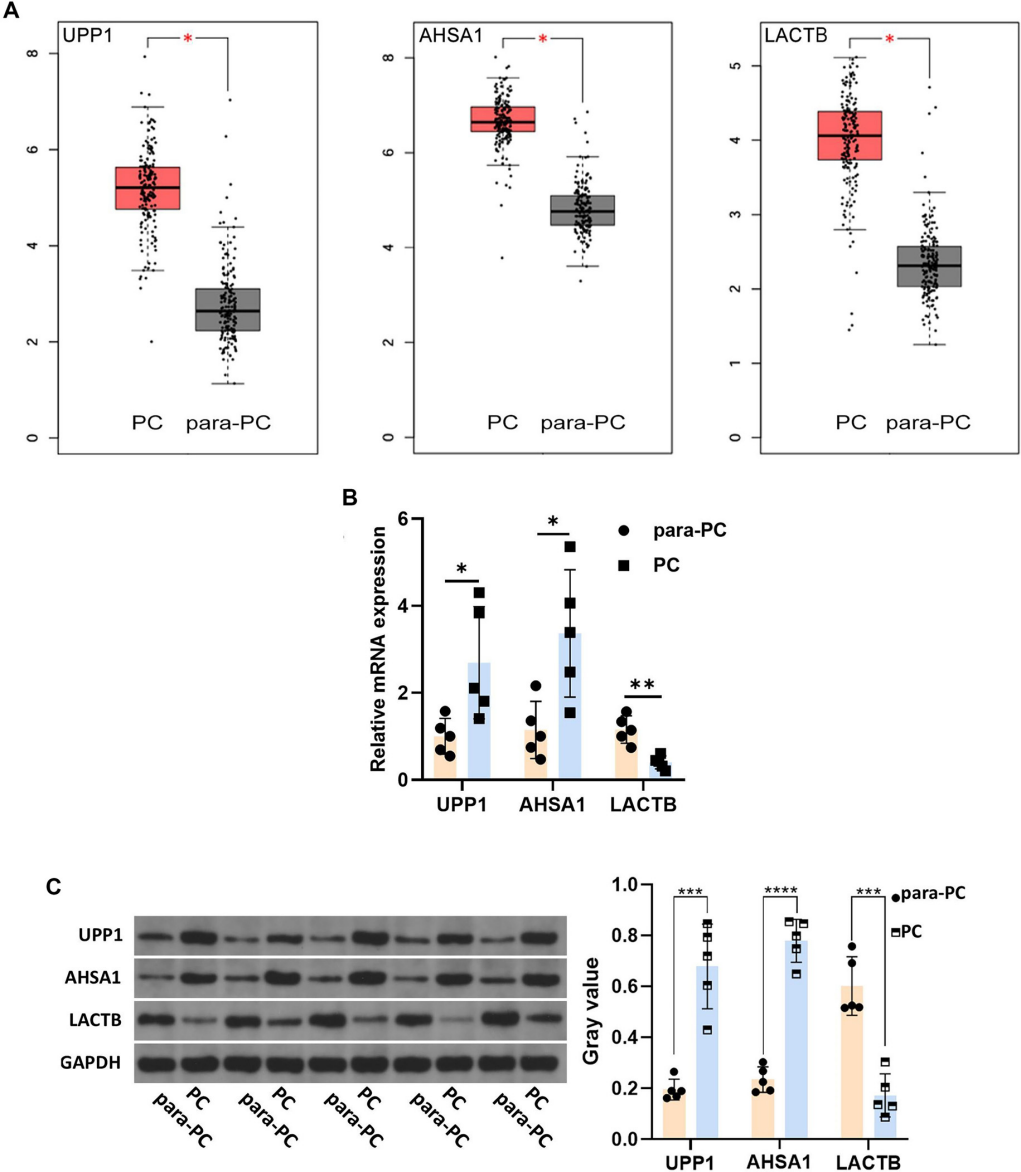
**GEPIA database (A), RT-qPCR (B), and Western blot (C) detected the expression of UPP1, AHSA1 and LACTB in PC vs para-PC group.** *P < 0.05, ***P < 0.001, ****P < 0.0001. PC: Pancreatic cancer; UPP1: Uridine Phosphorylase 1; LACTB: Lactamase Beta; AHSA1: Activator of HSP90 ATPase Activity 1; RT-qPCR: Reverse transcription-quantitative polymerase chain reaction; GEPIA: Gene Expression Profiling Interactive Analysis.

**Figure 10.**
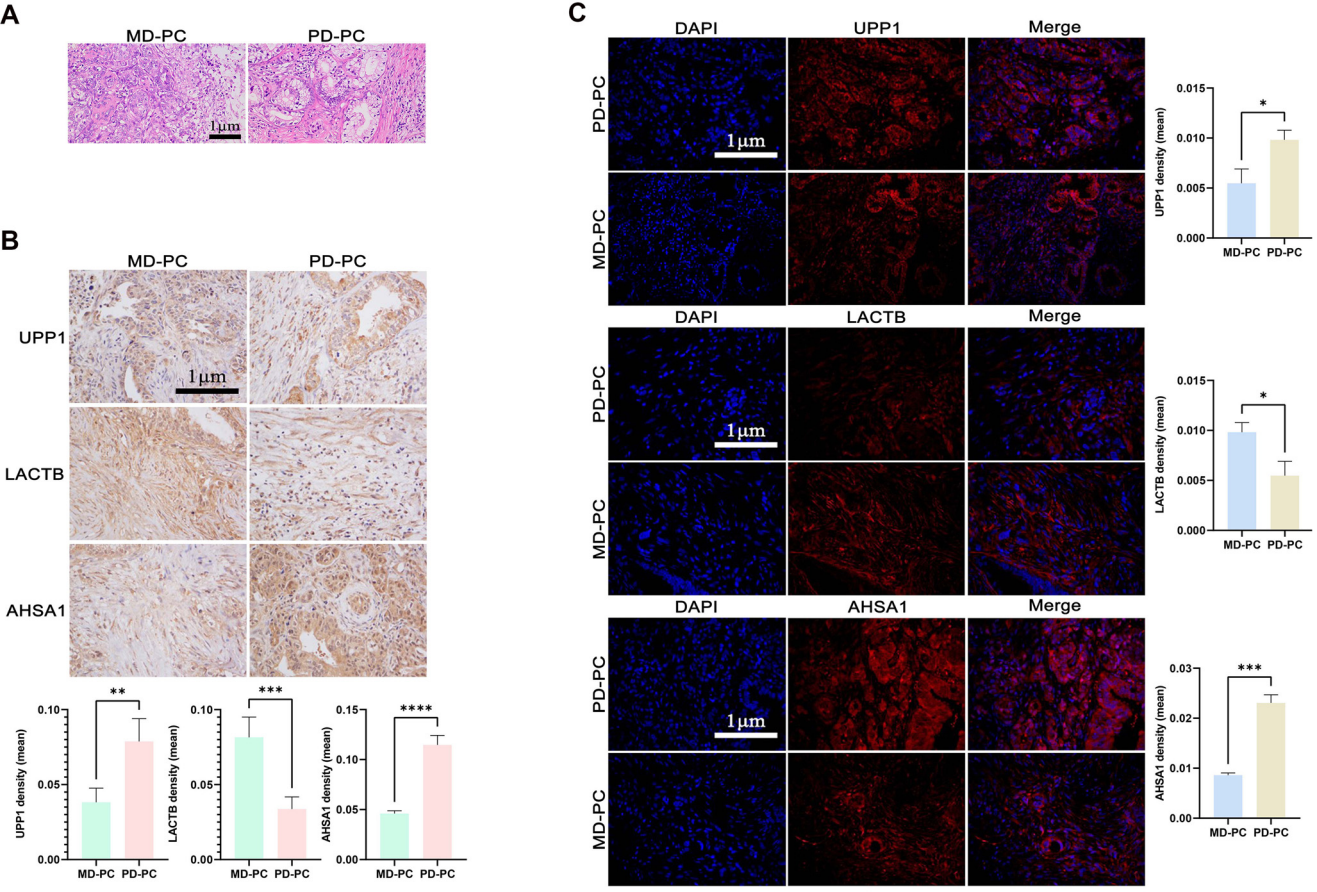
**H&E Staining (A), IF (B) and IHC (C) detected the expression of UPP1, AHSA1 and LACTB in PD-PC vs MD-PC group.** *P < 0.05, **P < 0.01, ***P < 0.001, ****P < 0.0001. UPP1: Uridine Phosphorylase 1; LACTB: Lactamase Beta; AHSA1: Activator of HSP90 ATPase Activity 1; H&E: Hematoxylin and eosin; IF: Immunofluorescence; IHC: Immunohistochemistry; PD: Poorly differentiated; PC: Pancreatic cancer; MD: Moderately differentiated.

The expression levels of target proteins in PC and para-PC groups were validated using the GEPIA database and Western blot experiments. HE, IF, and IHC staining were used to assess expression levels in PD-PC and MD-PC groups. As shown in Figure 9A, the GEPIA database indicated significantly lower expression levels of UPP1, AHSA1, and LACTB in the para-PC group compared to the PC group. RT-qPCR and Western blot analyses of five paired PC and adjacent normal tissues further revealed significantly elevated levels of UPP1 and AHSA1, and reduced levels of LACTB in PC samples (Figure 9A and 9B). To further validate UPP1, LACTB, and AHSA1 as key targets in PC development, we analyzed tissue samples from eight PD-PC and seven MD-PC cases. HE staining showed distinct histological features between PD and MD PCs: PD-PC cells exhibited high pleomorphism, with considerable variation in cell size and shape, the presence of duct-like structures, and abundant stromal fibrosis. Both groups displayed varying degrees of inflammatory infiltration (Figure 10A). IHC and IF experiments consistently showed higher average expression levels of UPP1 and AHSA1 in PD-PC tissues, while LACTB expression was higher in MD-PC tissues (Figure 10B and 10C), consistent with Western blot results. Although the LACTB expression trend differed from the database prediction, UPP1 and AHSA1 were rigorously validated as key targets in PC development and progression.

### Metabolic pathway and survival analyses of UPP1, LACTB, and AHSA1

**Figure 11.**
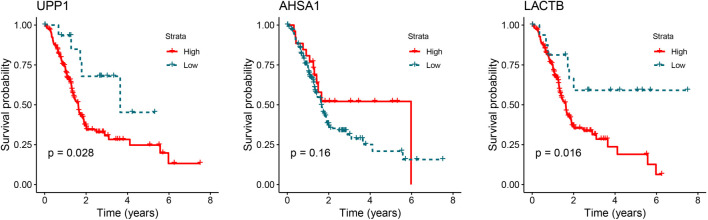
**Kaplan–Meier survival analysis.** UPP1: Uridine Phosphorylase 1; LACTB: Lactamase Beta; AHSA1: Activator of HSP90 ATPase Activity 1.

Based on our previous screening and preliminary verification, we identified UPP1 and AHSA1 as potential marker proteins for PC, exhibiting abnormally high expression in PC patients. Although pathway analysis has been conducted, the specific metabolic roles of UPP1, LACTB, and AHSA1 remain unclear. Moreover, their impact on PC patient survival outcomes warrants further investigation, particularly given that LACTB shows distinct alterations during PC progression compared to UPP1 and AHSA1. Leveraging KEGG analysis results, we focused on the metabolic pathways involving UPP1, LACTB, and AHSA1. Our findings revealed that only UPP1 is associated with the “Pyrimidine metabolism” and “Drug metabolism-other enzymes” pathways, where it is annotated as “2.4.2.3” (Figures S6 and S7). In the pyrimidine metabolism pathway, UPP1 catalyzes the conversion of uridine to uracil. Similarly, in the drug metabolism pathway, UPP1 facilitates the transformation of 5-fluoro-uridine into fluorouracil (5-FU). We conducted Kaplan–Meier survival analysis for UPP1, LACTB, and AHSA1 using the TCGA database (Figure 11). The results showed that patients with high expression levels of UPP1 and LACTB had significantly poorer overall survival compared to those with low expression levels.

## Discussion

In this study, we present a paradigm-shifting proteomic analysis of PC tissues, comparing PC with para-PC, as well as PD-PC with MD-PC. Our findings provide unprecedented insights into the molecular mechanisms underlying PC development and progression, and identify novel biomarkers and therapeutic targets with the potential to revolutionize PC management. Our comprehensive proteomic profiling reveals distinct molecular signatures associated with PC initiation (PC vs para-PC) and progression (PD-PC vs MD-PC), underscoring the complex and dynamic nature of PC evolution. These findings align with and significantly extend previous studies emphasizing the molecular heterogeneity of PC and its impact on disease advancement and treatment response [17, 18]. The identification of DEPs in both comparisons offers a more detailed and nuanced understanding of the proteomic changes driving PC, surpassing the scope of earlier reports [19, 20]. A key discovery of our study is the consistent upregulation of UPP1 and AHSA1 during both PC development and progression, validated across multiple experimental platforms. This finding suggests these proteins play pivotal roles in the disease process. UPP1, a key enzyme in the pyrimidine salvage pathway, may contribute to the altered nucleotide metabolism characteristic of cancer, thereby supporting rapid cellular proliferation. UPP1 is also known to be upregulated in several malignancies, including lung adenocarcinoma [21], bladder [22], gastric [23], and colorectal cancers [24]. AHSA1, an activator of heat shock protein 90 (HSP90), likely enhances the stability of multiple oncogenic proteins, promoting tumor growth and survival [25]. It is similarly overexpressed in hepatocellular carcinoma [26], breast cancer [27], and multiple myeloma [28]. We hypothesize that UPP1 facilitates metabolic reprogramming in PC cells, while AHSA1 may act as a master regulator of oncogenic signaling. These mechanistic roles establish a solid foundation for further functional studies and highlight both proteins as promising therapeutic targets. Functional enrichment analysis of DEPs revealed distinct pathway activations between the PC vs para-PC and PD-PC vs MD-PC comparisons, offering novel insights into the shifting biological landscape of PC. In the PC vs para-PC group, we observed significant enrichment in cell motility and lipid metabolism—processes critical for cancer cell survival and metastasis [29, 30]. In contrast, the PD-PC vs MD-PC comparison showed enrichment in immune response and coagulation pathways, reflecting a substantial shift in the tumor microenvironment as PC advances to a more aggressive phenotype. These findings support and expand on current research emphasizing the tumor microenvironment’s role in PC progression and its potential as a therapeutic target [31–33]. Further metabolic pathway analysis of UPP1, LACTB, and AHSA1 revealed that only UPP1 is directly involved in metabolism. UPP1 catalyzes the conversion of uridine to uracil, releasing ribose-1-phosphate, which can supply energy to proliferating cells. Given that 5-FU, a commonly used chemotherapeutic agent in PC, targets pyrimidine metabolism, UPP1 not only influences drug response but also supports the metabolic adaptation of cancer cells—positioning it as a dual therapeutic target. Our PPI network analysis further highlights functional differences across disease stages. In the PC vs para-PC network, key hub proteins, such as SRC, PTEN, and HDAC1—known regulators of cancer signaling and epigenetic control—were prominent [34, 35]. Meanwhile, immune-related proteins like C4B and A2M dominated the PD-PC vs MD-PC network, underscoring the growing role of immune modulation in advanced disease [36, 37]. This shift in network topology reflects the evolving molecular architecture of PC and could inform the design of stage-specific therapeutic strategies. Finally, Mfuzz clustering analysis revealed a progressive increase in the expression of proteins, including UPP1 and AHSA1 from para-PC to MD-PC to PD-PC. This trend provides compelling evidence of their involvement in PC progression and further supports their potential as biomarkers and therapeutic targets across disease stages [38, 39]. Their consistent upregulation suggests a fundamental role in PC pathogenesis, warranting deeper functional investigation.

The validation of our proteomic findings using the GEPIA database and experimental techniques strengthens the reliability and translational potential of our results. The consistent upregulation of UPP1 and AHSA1 across various experimental platforms and patient cohorts strongly supports their potential as robust biomarkers and therapeutic targets for PC [40]. Interestingly, we observed a discrepancy in LACTB expression between our experimental results and the GEPIA database. Since LACTB expression was not evaluated in databases beyond GEPIA, and our study only measured tissue transcription and translation levels without investigating post-transcriptional regulation, this unexpected result highlights the complex nature of protein expression regulation in neoplastic conditions and underscores the need for further research into LACTB’s role in PC progression. We hypothesize that this discrepancy could result from post-transcriptional regulatory mechanisms or tissue-specific effects. This finding underscores the importance of employing multiple validation approaches in proteomic studies and opens new avenues for investigating LACTB regulation in PC [41]. Regrettably, the GEPIA database lacks data from healthy individuals. Consequently, direct comparisons of UPP1, LACTB, and AHSA1 expression between healthy individuals and PC patients will be addressed in future validation studies. The identification of stage-specific protein expression patterns and associated pathways offers a novel framework for understanding PC progression and may inform personalized treatment strategies. This aligns with the growing emphasis on precision medicine in cancer treatment, where molecular profiling is used to tailor therapies to individual patients [8, 42]. Our findings contribute to this paradigm by providing a detailed proteomic landscape that could guide the development of targeted therapies and personalized regimens. Our study has significant clinical implications. Using Kaplan–Meier survival analysis, we found that high expression of UPP1 and LACTB is associated with poor survival outcomes. The identification of UPP1 and AHSA1 as potential biomarkers may facilitate early detection of PC—addressing a major challenge in managing the disease [43]. These proteins could potentially be developed into blood-based biomarkers for non-invasive PC screening. Moreover, they may serve as therapeutic targets, enabling the development of novel treatment strategies for PC. For example, small molecule inhibitors targeting UPP1 or AHSA1 could be explored as potential therapies. However, translating these findings into clinical applications will require extensive validation in larger cohorts, along with functional studies to clarify the biological roles of these proteins in PC [44]. Despite these promising findings, we acknowledge several limitations of our study. To ensure that research findings reflect disease-specific differences rather than inter-individual variability or environmental factors, we primarily focused on protein expression differences between tumor tissue and adjacent non-tumor tissue. However, some molecular alterations may be shared by both tissue types. For instance, the discrepancy in LACTB expression trends between our experimental data and the GEPIA database may stem from differences between adjacent non-tumor tissue and truly healthy pancreatic tissue, highlighting the need for future validation using appropriate healthy controls. While our sample size is adequate for an initial proteomic investigation, larger cohort studies will be necessary to confirm the clinical relevance of our findings—particularly the inconsistent LACTB expression patterns observed between datasets. Additionally, although we identified candidate biomarkers and therapeutic targets, functional studies are required to elucidate their precise roles in PC biology. Our study focused primarily on protein expression levels, and future research should incorporate analyses of post-translational modifications and PPIs to provide a more comprehensive view of the PC proteome [45]. We also recognize that intratumoral heterogeneity was not addressed in our analysis, which could affect proteomic profiles. Future studies should explore the mechanistic roles of UPP1 and AHSA1 in PC using laboratory and animal models. Furthermore, integrating proteomic data with gene expression and small molecule research will be essential to gaining a deeper understanding of pancreatic cancer biology.

## Conclusion

In conclusion, our comprehensive proteomic analysis offers novel insights into the molecular landscape of PC progression, marking a significant advancement in the field. The identification of UPP1 and AHSA1 as key contributors highlights new opportunities for biomarker discovery and therapeutic development. These findings not only deepen the current understanding of PC proteomics but also lay a strong foundation for future research.

## Supplemental data

Supplemental data are available at the following link:

https://www.bjbms.org/ojs/index.php/bjbms/article/view/11958/3872

https://www.bjbms.org/ojs/index.php/bjbms/article/view/11958/3873

https://www.bjbms.org/ojs/index.php/bjbms/article/view/11958/3876

## Data Availability

The data that support the findings of this study are available from https://www.iprox.cn/. (Accession number: IPX0011770000).
